# Bone Marrow Mesenchymal Stem Cell-Derived Exosome-Educated Macrophages Promote Functional Healing After Spinal Cord Injury

**DOI:** 10.3389/fncel.2021.725573

**Published:** 2021-09-28

**Authors:** Chengjun Li, Tian Qin, Jinyun Zhao, Rundong He, Haicheng Wen, Chunyue Duan, Hongbin Lu, Yong Cao, Jianzhong Hu

**Affiliations:** ^1^Department of Spine Surgery and Orthopaedics, Xiangya Hospital, Central South University, Changsha, China; ^2^National Clinical Research Center for Geriatric Disorders, Xiangya Hospital, Central South University, Changsha, China; ^3^Key Laboratory of Organ Injury, Aging and Regenerative Medicine of Hunan Province, Changsha, China; ^4^Department of Sports Medicine, Research Centre of Sports Medicine, Xiangya Hospital, Central South University, Changsha, China

**Keywords:** exosome-educated macrophages, spinal cord injury, neurovascular unit, angiogenesis, axon growth

## Abstract

The spinal cord injury is a site of severe central nervous system (CNS) trauma and disease without an effective treatment strategy. Neurovascular injuries occur spontaneously following spinal cord injury (SCI), leading to irreversible loss of motor and sensory function. Bone marrow mesenchymal stem cell (BMSC)–derived exosome-educated macrophages (EEM) have great characteristics as therapeutic candidates for SCI treatment. It remains unknown whether EEM could promote functional healing after SCI. The effect of EEM on neurovascular regeneration after SCI needs to be further explored. We generated M2-like macrophages using exosomes isolated from BMSCs, which were known as EEM, and directly used these EEM for SCI treatment. We aimed to investigate the effects of EEM using a spinal cord contusive injury mouse model *in vivo* combined with an *in vitro* cell functional assay and compared the results to those of a normal spinal cord without any biological intervention, or PBS treatment or macrophage alone (MQ). Neurological function measurements and histochemical tests were performed to evaluate the effect of EEM on angiogenesis and axon regrowth. In the current study, we found that treatment with EEM effectively promoted the angiogenic activity of HUVECs and axonal growth in cortical neurons. Furthermore, exogenous administration of EEM directly into the injured spinal cord could promote neurological functional healing by modulating angiogenesis and axon growth. EEM treatment could provide a novel strategy to promote healing after SCI and various other neurovascular injury disorders.

## Introduction

Spinal cord injury (SCI) is a type of severe central nervous system (CNS) trauma that usually leads to permanent neurological deficits (van Middendorp et al., 2011; GBD 2016 Neurology Collaborators, 2019). SCI is accompanied by complex pathological events and often restricts the regeneration of nervous tissue at the injury site (Fan et al., 2018; Albayar et al., 2019). Acute trauma damages not only vascular tissue but also neuroparenchymal tissue, including neurons and oligodendrocytes (Fan et al., 2018). Neurovascular injuries occur spontaneously following SCI and frequently lead to the irreversible loss of motor and sensory function (White and El-Khoury, 2002). Successful neurological functional reconstruction requires blood supply to improve nerve regeneration capacity (Han et al., 2010). However, therapy to promote angiogenesis is usually insufficient for neuroprotection after SCI. Additional attention should be given to enhancing neurogenesis in acute trauma and during the repair phase (Li X. et al., 2020). Therefore, an ideal restorative approach to enhancing long-term functional recovery after SCI should promote both postinjury axon growth and vascular perfusion in the injured region. A great number of methods have been developed as treatment options for SCI that promote angiogenesis coupled to axon growth (Li X. et al., 2020; Liu et al., 2021).

Macrophages are emerging as key players and have crucial roles in tissue repair and homeostasis (Gensel and Zhang, 2015). Macrophages are highly plastic cells; under different conditions, these cells can be polarized into different subsets, including M1 (classically activated) and M2 (alternatively activated) macrophages (Xie et al., 2019). Macrophage polarization can be induced by the paracrine activity of mesenchymal stem cells (MSCs) (Xin et al., 2020). Currently, the use of macrophages after coculture with exosomes derived from MSCs to produce MSC-derived exosome-educated macrophages (EEM) has greatly attracted attention as a therapeutic strategy in the regeneration phase. Several experimental injury models have demonstrated the protective effect of BMSC-derived EEM. Research has revealed that EEM can modulate the remodeling process and improve ligament healing (Chamberlain et al., 2021). It has also been reported that treatment with EEM can enhance the healing of the Achilles tendon (Chamberlain et al., 2019). In addition, treatment with EEM could relieve acute radiation syndrome by promoting hematopoietic recovery (Kink et al., 2019). These findings suggest that EEM are completely different from naïve macrophages and can be regarded as a novel type of alternatively activated macrophage that has many novel features. Thus, in our current study, we demonstrated that EEM transplantation facilitated neurological functional recovery in association with promoting angiogenesis and axon growth after SCI.

## Materials and Methods

### Isolation and Characterization of Exosomes Derived From Bone Morrow Mesenchymal Stem Cells

Bone morrow mesenchymal stem cells (BMSCs) were isolated from the bone marrow of mouse femurs as previously described (Boregowda et al., 2016). BMSCs were identified by using osteogenic, adipogenic, and chondrogenic differentiation media to confirm their multipotent differentiation potential. BMSCs were characterized by flow cytometry after being incubated with antibodies against specific surface antigens, including CD45, CD34, CD11b, CD29, CD90, and Sca-1 (BD Biosciences. San Jose, CA, United States) according to the manufacturer’s recommendations. Final quantification was conducted with a BD FACSAria III flow cytometer (BD Biosciences). Stained BMSCs were analyzed and compared to the corresponding unstained cells. The data were analyzed using flow cytometry data analysis software (FlowJo V10, FlowJo, LLC, Ashland, OR, United States). Exosomes were isolated from BMSCs at passages 3–5 using an Optima^TM^ L-80XP Ultracentrifuge (Beckman Coulter). Exosome concentrations and diameters were measured using IZON qNano Nanoparticle (Zen-Bio, Inc., Research Triangle Park, North Carolina) analysis. Exosome protein and RNA concentrations were analyzed using a NanoDrop spectrophotometer (Thermo-Fisher Scientific, Waltham, MA, United States). The exosomes were visualized by transmission electron microscopy (TEM).

### Macrophage Generation and Education by Exosomes Derived From Bone Morrow Mesenchymal Stem Cells

Mouse bone marrow–derived macrophages (BMDMs) were isolated as previously described (Manzanero, 2012). Bone marrow cells were cultured in DMEM containing 10% FBS, and 50 ng/ml M-CSF was added for 3 days to obtain BMDMs. To generate EEM, 1 × 10^6^ BMDMs were supplemented with exosome-free media and treated with 200 μl of exosomes derived from BMSCs for 3 days. Macrophages treated with the same amount of PBS were designated control macrophages (MQ).

### Macrophage Polarization Assay

Exosomes were labeled with PKH26 (Invitrogen, Eugene, OR) for *in vitro* cell tracking. Exosomes or PBS was incubated with BMDMs and stained with DAPI (Vectashield, Vector Laboratories, Burlingame, CA, United States) and F4/80 surface markers to visualize exosome internalization using a fluorescence microscope (BIOREVO BZ7000; Keyence; Osaka, Japan). After 3 days of culture, macrophages or EEM were collected, blocked with 3% FBS, and then incubated with PerCP Cy5.5-conjugated anti-mouse CD11b (1:200, BioLegend, San Diego, CA), APC-conjugated anti-mouse F4/80 (1:200, BioLegend, San Diego, CA), FITC-conjugated anti-mouse CD86 (1:200, BioLegend, San Diego, CA), and PE-conjugated anti-mouse CD206 (1:200, BioLegend, San Diego, CA) antibodies according to the manufacturer’s instructions and analyzed using a BD FACSAria III flow cytometer (BD Biosciences). Unstained macrophages were used as the negative control. Total RNA was extracted from macrophages after different treatments using the RiboEx reagent. Quantitative real-time PCR was performed according to previously described methods using LightCycler 480 SYBR Green I Master mix (Roche Molecular Systems, Pleasanton, CA, United States) on a LightCycler 480 System (Roche) under the following conditions: 95°C for 5 min and 95°C for 10 s, 45 cycles of 60°C for 20 s, and 72°C for 15 s. The primer sequences used in the current study are listed in Table 1. Gene expression was normalized to that of GAPDH.

**TABLE 1:** Primers for qPCR. (5′–3′).

iNOS-F	GTTCTCAGCCCAACAATACAAGA
iNOS-R	GTGGACGGGTCGATGTCAC
TNF-α-F	GCTCCTCCACTTGGTGGTTT
TNF-α-R	AGGCGGTGCCTATGTCTCAG
CCR7- F	TGTACGAGTCGGTGTGCTTC
CCR7-R	GGTAGGTATCCGTCATGGTCTTG
Arginase-F	GAGCCACCGTTTTACATTGTGA
Arginase- R	CTCGCCCACTAGGCAGTTC
IL-10-F	GCTCTTACTGACTGGCATGAG
IL-10-R	CGCAGCTCTAGGAGCATGTG
CD206-F	AGCTTCATCTTCGGGCCTTTG
CD206-R	GGTGACCACTCCTGCTGCTTTAG
GAPDH-F	AGGTCGGTGTGAACGGATTTG
GAPDH-R	TGTAGACCATGTAGTTGAGGTCA

### Scratch Wound Migration and Tube Formation Assay

To investigate the effect of EEM and MQ on human umbilical vein endothelial cells (HUVECs), macrophage-conditioned medium (CM) was collected and cultured with HUVECs for migration analysis. Briefly, 3 days after exosomes or PBS were added, the culture medium was removed and the EEM or MQ was washed with PBS to eliminate BMSC-derived exosomes. The EEM or MQ was added with new macrophage cell culture medium (exosome-free), and continued to culture for 3 days. Then, we collected the culture medium (CM) of EEM and MQ. HUVECs were cultured in six-well plates and treated with 200 μl of CM from different macrophage cultures (1.3 ml of HUVEC culture medium was also added to each well). HUVECs grew at to 90% confluence, and a 200-μl pipette tip was used to scrape a cross shape in the monolayer. Images were captured at 0 and 12 h after treatment with 200 μl of CM using a microscope (CKX41; Olympus Corporation, Tokyo, Japan); the wound width was recorded as previously described (Ni et al., 2019). Capillary network formation was evaluated using an *in vitro* angiogenesis assay tube formation kit (Cultrex, United States) according to the manufacturer’s instructions. HUVECs (2 × 10^4^ cells/well) were plated on Matrigel (100 μl/well) and treated with 25 μl of CM from different cultured macrophages. The migration distance, total tube numbers, and branch points were measured using Image-Pro Plus 7.0 software in a blinded manner.

### CCK-8 Assay

CCK-8 assay was used to detect the proliferation of HUVECs. Firstly, we adjusted the cell concentration to 50,000/ml, and added 100 μl of cell suspension to each well of a 96-well plate. HUVECs were treated with 25 μl EEM-CM or macrophage-CM. After pre-culture (5% CO_2_, 37°C) for 24, 48, 72, or 96 h, change the culture medium added with 10 μl CCK-8 solution (MCE, China) and continue the culture for 2 h. The absorbance at 450 nm was measured with a microplate reader (Thermo Fisher Scientific, United States).

### Cortical Neuron Axonal Growth Assay

Primary cortical neurons were isolated from the cerebral cortex of E16 embryonic C57BL/6 mouse pups according to previously described methods (Kim and Magrané, 2011). The harvested cells were resuspended and pre-purified by density gradient separation to remove astrocytes, oligodendrocytes, and microglia and with the purity of more than 95% and culture with neurobasal medium (Cyagen, United States) containing 2% B27, 1% glutamine, and 1% penicillin-streptomycin in six-well plates. Then the primary cortical neurons were incubated with 200 μl of CM from different macrophage cultures (1.3 ml of neuron culture medium was also added to each well). After 48 h of culture, the cells were fixed and stained with anti-Tuj-1 (1:400, Abcam). Stained images were visualized under a fluorescence microscope (Carl Zeiss Axio Observer Z1, Oberkochen, Germany), and neuronal length was measured using Image-Pro Plus 7.0 software in a blinded manner.

### Animals and Spinal Cord Contusive Injury Model

Female C57BL/6 mice (8 weeks old) were used in this study. All experimental procedures were performed strictly in accordance with the Animal Care and Use Guidelines and approved by the Animal Ethics Committee of Central South University, Changsha, China. All surgical procedures were performed using a sublethal injection of 8 mg/kg ketamine and 10 mg/kg xylazine for anesthesia to minimize animal suffering. Female mice (8 weeks old) were used as an animal model to study healing after SCI in response to treatment with EEM, MQ, or the same amount of PBS. Following anesthesia, the animals underwent thoracic laminectomy and were subjected to a contusion injury (50 kdyne) at the T10 level of the spine using an Infinite Horizon’s impactor (Precision Systems Instrumentation) (Park et al., 2016). After generating the SCI model, the bladders of the mice were manually pressed three times daily during the first week after surgery.

### Macrophage Transplantation and *in vivo* Imaging

PBS-treated macrophages and EEM were labeled with DiR (Invitrogen) or PKH26 (Sigma). The hydrogel composed of 5% methacrylated gelatin (GelMA) and 1.25% N-(2-aminothyl)-4-(4-(hydroxymethyl)-2-methoxy-5-nitrosophe-noxy) butanamide (NB) linked to the glycosaminoglycan hyaluronic acid (HA-NB) with 0.1% of the polymerization initiator lithium phenyl-2.4.6-trimethylbenzoylphosphinate (LAP). After ultraviolet irradiation, GelMA quickly formed the first cross-link network of the hydrogel. The aldehyde groups on HA-NB react with the amino groups of GelMA and tissues to form a second cross-link network, finally forming a gel (Hong et al., 2019). After SCI, we directly resuspended the 510^4^ labeled EEM or MQ with liquid hydrogel (after mixing, the cells are evenly distributed in the hydrogel) and then applied the hydrogel containing EEM or MQ on the surface of the injured spinal cord, and irradiated with ultraviolet light for 5–8 s. Finally, the hydrogel appeared to be a stable gel on the surface of the injured site of the spinal cord. For *in vivo* imaging, the mice treated with DiR-labeled EEM or MQ were anesthetized at 7 days after transplantation and placed in a Xenogen IVIS Imaging System (Caliper Life Sciences). The mice treated with PKH26-labeled macrophages or EEM were sacrificed at 7 days post-SCI and spinal cord was harvested for frozen sectioning and Co-staining wit F4/80 (1:400, Abcam) and PKH26 to detect macrophage distribution. For *in vivo* analysis of survival MQ and EEM, after 7 days of transplantation, the injury spinal cords were collected. Followed by digestion *via* 0.125% Trypsin-EDTA (Gibco, United States), the resulting cell suspensions were filtered (40 μm) and washed using PBS containing 1% FBS. After washing, cells were blocked with 3% BSA and then incubated with FITC-conjugated anti-mouse F4/80 (1:200, BioLegend, San Diego, CA) antibodies for 45 min on ice. After washing, cells were resuspended in PBS (pH 7.2) with 1 μg/ml DAPI (BD Biosciences, United States) for live/dead exclusion and analyzed by BD FACSAria III flow cytometer (BD Biosciences, United States).

### Behavioral Assessments and Electrophysiology Testing

BMS score testing was performed to evaluate locomotor functional recovery after SCI. Briefly, mice were placed in an open field. Two trained experimenters who were blinded to the experimental groups evaluated the BMS scores on a scale of 0–9 (0, complete hind limb paralysis; 9, normal locomotion) on days 1, 3, and 7 and once per week thereafter for 8 weeks post SCI. A von Frey filament test was performed by using Semmes-Weinstein monofilaments (Stoetling Company, Wooddale, IL, United States) with forces ranging from 0.008 to 300 N as described previously. Tests were performed three times with an interval of 10 min before model induction and on days 1, 3, and 7 and once per week thereafter for 8 weeks post SCI. The hindlimb motor evoked potential (MEP) and latent period were obtained as previously described (Guo et al., 2021). Briefly, recording needle electrodes were inserted intramuscularly into the tibialis anterior muscle of the hindlimbs. The stimulating electrodes were placed subcutaneously corresponding to the cortical motor area. Reference electrode was inserted under the skin of the back. The average latency and amplitude of the MEP values were acquired before SCI surgery and at 56 days after SCI treatment with different intervention.

### 3D Neurovascular Visualization and Quantification Using Synchrotron Radiation Micro-Tomography

Synchrotron radiation micro-tomography (SRμCT) is a non-destructive three-dimensional (3D) imaging technique that can produce large area collimated beams for high flux x-ray imaging over a much larger field of view (FOV) than conventional method. Visualization of neural network and vasculature was performed separately. For neural network visualization, mice were anesthetized and perfused transcranially with artificial cerebrospinal fluid to remove the blood from the vessels. The spinal cord at the injured site was isolated and processed by staining with Golgi-Cox solutions in a FD Rapid GolgiStain^TM^ (KitNeuro Technologies, Inc.) as previously described (Jiang et al., 2021a). Briefly, after Golgi-Cox staining, the spinal cord was prepared for SRμCT analysis. For vascular imaging, mice were anesthetized and perfused transcardially with heparin saline, followed by the low-viscosity radio opaque polymer Microfill (Flow Tech, Inc., Carver, MA, United States) for vessel visualization using SRμCT.

The spinal cord specimens were scanned with a BL13W1 beamline at the Synchrotron Radiation Facility (SRF) using SRμCT. The image parameters were set with a physical pixel size of 1.625 μm × 1.625 μm. After the samples were rotated during scanning, 900 projection images were captured and transformed into 2D slice images with PITRE software developed by the SSRF as previously described (Cao et al., 2019). Then, all 2D slices were reconstructed into 3D images using Amira software (Version 6.01, FEI, United States). The vascular morphology and neuro network were extracted using gray value base segmentation. For quantitative analysis of the morphological parameters of the neural network, a region of interest (ROI) of 225 μm × 225 μm × 225 μm volume was selected from the anterior horn of the spinal cord at the 500 μm rostrally adjacent to the injured epicenter. For vascular morphological parameters analysis, a ROI of 500 μm × 500 μm × 1,000 μm volume of spinal cord tissue including the injured epicenter was selected for analysis. The vessel volume fraction (VVF), vessel segment density (VSD), bifurcation point density (BPD), neural soma volume (NSV), and axon length (AL) were systematically analyzed by measuring the number of pixels belonging to each category with Image-Pro Analyzer 3D (Version 7.0, Media Cybernetics, Inc., Bethesda, MD, United States) as previously described (Cao et al., 2017; Jiang et al., 2021b).

### Histopathological Immunofluorescence Examination

Following the final image acquisition, the animals were sacrificed, and the spinal cords were collected and prepared for histopathological examination. The spinal cords were placed in optimal cutting tissue medium and transversely sectioned at a thickness of 16μm. Representative sections of 200 μm around the injury epicenter of the spinal cord were chosen for immunological staining, and spinal sections on slides were incubated with anti-CD31 (1:200, R&D), anti-Ki67 (1:400, Abcam), anti-CSPG (1:400, Sigma), and anti-Tuj-1 (1:400, Abcam) primary antibodies, after which they were incubated with the respective secondary antibodies to visualize different color emissions. After being washed, the sections were mounted with Vectashield DAPI Hardmount (Vector Laboratories) and were examined under a fluorescence microscope (Carl Zeiss Axio Observer Z1, Oberkochen, Germany).

### Statistical Analysis

The results were statistically analyzed using GraphPad Prism software (version 7.00, La Jolla, California, United States). All data are presented as the means ± standard deviation (SD). Unpaired *t*-test was used to analyze differences between two groups. Comparisons among different groups were performed by one-way ANOVA followed by Tukey’s *post hoc* test. For BMS score, tactile sensory test, and CCK-8 analysis, repeated two-way ANOVA followed by Tukey’s *post hoc* test was performed. *p*-values less than 0.05 were considered statistically significant.

## Results

### The Identification of Mouse Bone Morrow Mesenchymal Stem Cells and Bone Marrow Mesenchymal Stem Cell-Derived Exosomes

**FIGURE 1:**
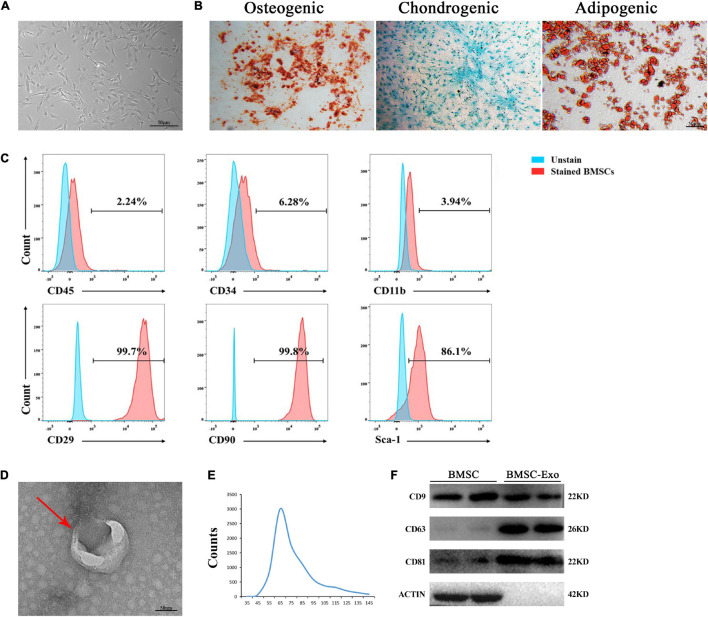
Morphology and characterization of BMSCs and BMSC-derived exosomes. **(A)** Representative microscopic image of BMSCs. Scale bar: 50 μm. **(B)** BMSCs demonstrated osteogenic, chondrogenic, and audiogenic pluripotent differentiation abilities. Scale bar: 50 μm. **(C)** Flow cytometry analysis of cell surface markers on BMSCs. **(D)** Transmission electron microscopy (TEM) image of BMSC-derived exosomes. Scale bar: 50 nm. **(E)** NanoSight tracking analysis (NTA) showed that the average diameters of exosomes ranged from 30 to 150 nm. **(F)** Western blot analysis of Tsg101 CD9, CD63, and actin.

The BMSCs exhibited a spindle-like morphology (Figure 1A) and could differentiate into osteoblasts, adipocytes, or chondrocytes, as evaluated by Alizarin Red S staining, Oil Red O staining, and Alcian Blue staining, respectively (Figure 1B). BMSCs highly expressed the stem cell markers CD29, CD90, and Sca-1 but had negative expression of CD45, CD34, and CD11b, as revealed by flow cytometry (Figure 1C). Exosomes derived from BMSCs were round-shaped in morphology with a bilayer membrane structure, as observed by TEM (Figure 1D). The isolated exosomes showed vesicle sizes ranging from 30 to 150 nm and with a concentration of approximately 1–2 × 10^10^ particles/ml (Figure 1E). Western blotting demonstrated that BMSC-derived exosomes highly expressed exosome-specific protein markers (TSG101, CD9, and CD63), but not actin (Figure 1F).

### The Characteristics of Exosome-Educated Macrophages

**FIGURE 2:**
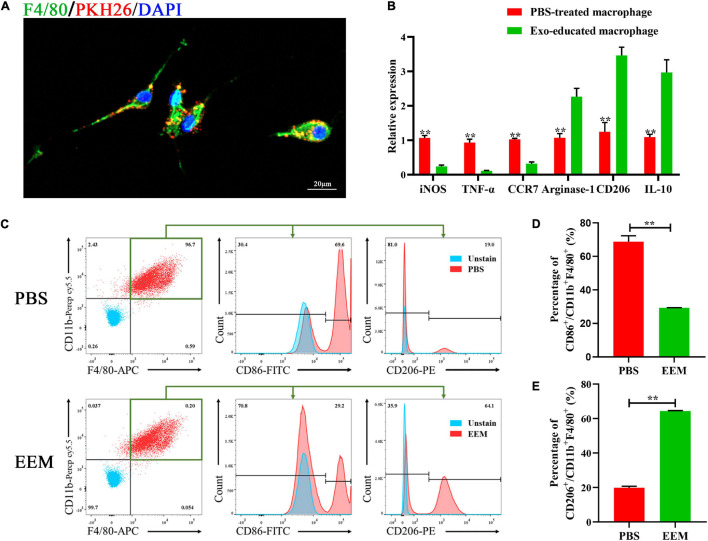
Exosomes educate macrophages to become M2-like macrophages. **(A)** Analysis of cellular exosome uptake by macrophages. PKH26: red, DAPI: blue, F4/80: green. Scale bar: 20 μm. **(B)** Macrophage mRNA was analyzed for the indicated M1 or M2 marker genes by RT-PCR (iNOS: *t* = 17.45, *p* < 0.01; TNF-α: *t* = 14.19, *p* < 0.01; CCR7: *t* = 21.55, *p* < 0.01; Argnase-1: *t* = 7.714, *p* < 0.01; CD206: *t* = 10.61, *p* < 0.01; IL-10: *t* = 8.737, *p* < 0.01; *n* = 3 biological replicates for each group. **(C)** Changes in surface antigen expression in educated macrophages was analyzed by flow cytometry. **(D)** Quantitation of percentage of CD86^+^/CD11b^+^F4/80^+^ of **(C)** (*t* = 19.39, *p* < 0.01, *n* = 3 biological replicates for each group). **(E)** Quantitation of percentage of CD206^+^/CD11b^+^F4/80^+^ of **(C)** (*t* = 80.15, *p* < 0.01, *n* = 3 biological replicates for each group). The data are presented as the means ± SD, *p < 0.05, **p < 0.01, NS = Not significant.

Next, we used PKH26 to label exosomes and applied the labeled exosomes to educate macrophages. BMSC-derived exosomes (BMSC-Exos) or PBS were added to the culture medium of macrophages. After the macrophages were incubated with PKH26-labeled exosomes for 24 h, the PKH26-labeled exosomes in the cytoplasm were observed by fluorescence microscopy to visualize the cellular uptake of PKH26-labeled exosomes (Figure 2A). As shown in Figure 2B, the mRNA expression levels of iNOS, TNF-α, and CCR7 (M1 macrophage markers) were significantly downregulated in the EEM treatment groups. Arginase-1, IL-10, and CD206 (M2 macrophage markers) were increased after treatment with exosomes derived from BMSCs. In addition, we examined the expression of CD206 (a marker of M2 macrophages) and CD86 (a marker of M1 macrophages) in treated macrophages using flow cytometry. As shown in Figure 2C, the expression level of CD86 was significantly higher in the PBS group than the exosome treatment group, while the expression level of CD206 was higher in EEM-treated macrophages than in PBS-treated macrophages (Figures 2D,E). These data indicated that BMSC-derived exosomes were able to educate macrophages to become M2-like macrophages.

### Exosome-Educated Macrophages Promote Angiogenic Activity and Axon Growth *in vitro*

**FIGURE 3:**
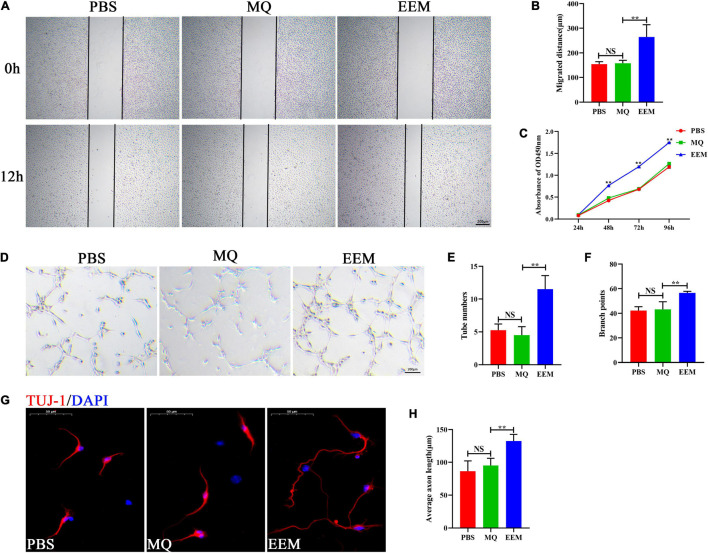
EEM promote angiogenic activity and axon growth *in vitro*. **(A)** Representative images of the horizontal migration of HUVECs in the EEM, MQ, and PBS treatment groups, as shown by wound healing experiments. **(B)** Quantification of the migration distance shown in **(A)** [*F*(2, 9) = 17.75, *p* < 0.05. Tukey’s *post hoc* test. PBS vs. MQ, *p* = 0.98; MQ vs. EEM, *p* < 0.01; *n* = 4 biological replicates for each group]. **(C)** Analysis of HUVECs proliferation by CCK-8 [*F*(2, 6) = 1,346, *p* < 0.01. Tukey’s *post hoc* test. MQ vs. EEM (24 h), *p* = 0.5071; MQ vs. EEM (48 h), *p* < 0.01; MQ vs. EEM (72 h), *p* < 0.01; MQ vs. EEM (96 h), *p* < 0.01; *n* = 3 biological replicates for each group]. **(D)** Representative images of HUVEC canaliculization in the EEM, MQ, and PBS treatment groups. Scale bar: 200 μm. **(E)** Quantification of tube numbers of **(D)** [*F*(2, 9) = 25.63, *p* < 0.01. Tukey’s *post hoc* test. PBS vs. MQ, *p* = 0.77; MQ vs. EEM, *p* < 0.01; *n* = 4 biological replicates for each group]. **(F)** Quantification of branch points of **(D)** [*F*(2, 9) = 15.98, *p* < 0.01. Tukey’s *post hoc* test. PBS vs. MQ, *p* = 0.93; MQ vs. EEM, *p* < 0.01; *n* = 4 biological replicates for each group]. **(G)** Representative immunofluorescence images of TUJ1 (red) in the EEM, MQ, and PBS treatment groups. Scale bar: 50 μm. **(H)** Quantification of the average axon length in **(G)** [*F*(2, 12) = 19.03, *p* < 0.01. Tukey’s *post hoc* test. PBS vs. MQ, *p* = 0.53; MQ vs. EEM, *p* < 0.01; *n* = 5 biological replicates for each group]. The data are presented as the means ± SD. *p < 0.05, **p < 0.01, NS = Not significant.

To investigate the effect of EEM and MQ on angiogenesis and axon growth, HUVECs and primary cortical neurons were selected and used for our current investigation. Scratch wound healing assays demonstrated that EEM significantly promoted the horizontal migration of HUVECs compared with those in the PBS group; however, treating with macrophages culture medium exhibited no effect on HUVEC migration (Figures 3A,B). CCK-8 assay was used to evaluate the proliferation of HUVECs. As shown in Figure 3C, HUVECs treated with EEM culture medium showed stronger proliferation ability than that with macrophage culture medium or PBS. Furthermore, tube formation assays on Matrigel revealed that the canaliculization of HUVECs was significantly greater in the EEM treatment group than the control group, as indicated by the increased numbers of total branching points and tubes than in the PBS treatment group (Figures 3D–F). Then, we assessed whether EEM could also stimulate neurite extension in primary cortical neurons, and the results revealed that EEM could significantly promote axonal growth compared with that in the MQ treatment group or control group. There was no difference in axon growth between the PBS and MQ treatment groups (Figures 3G,H).

### Exosome-Educated Macrophages Promote Neurological Functional Recovery After Spinal Cord Injury

**FIGURE 4:**
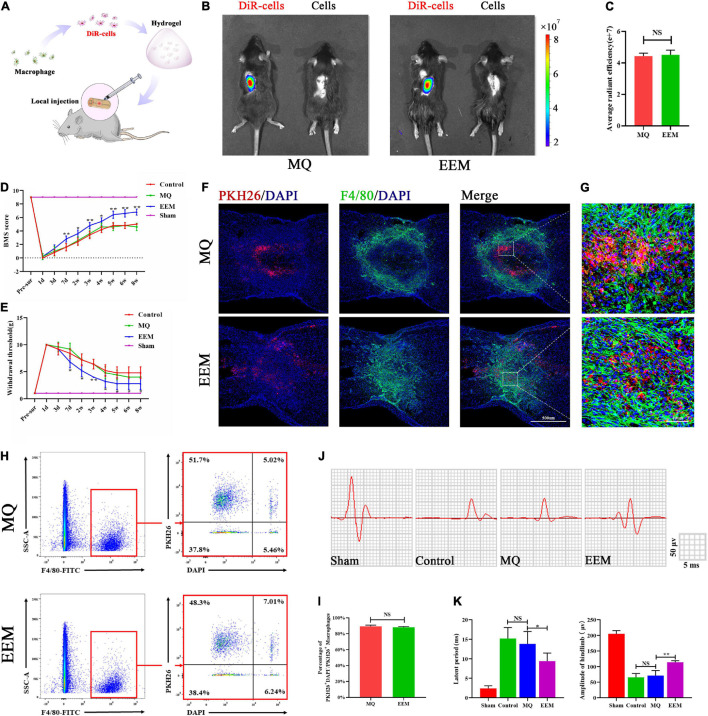
EEM promote sensory and motor functional recovery after SCI. **(A)** Schematic depiction of the approaches used to transplant the DiR-labeled EEM or MQ. **(B)** *In vivo* tracing of the distribution of DiR-labeled EEM and MQ embedded in hydrogels in the injured spinal cord at 3 days. **(C)** Quantification of average fluorescence efficiency of **(B)** (*t* = 0.40, *p* = 0.54, *n* = 3 animals per group). **(D)** BMS scores after SCI in the different treatment groups [*F*(3, 16) = 586.4, *p* < 0.01. Tukey’s *post hoc* test. MQ vs. EEM (1d), *p* = 0.99; MQ vs. EEM (3d), *p* = 0.75; MQ vs. EEM (7d), *p* < 0.01; MQ vs. EEM (2w), *p* < 0.05; MQ vs. EEM (3w), *p* < 0.01; MQ vs. EEM (4w), *p* < 0.05; MQ vs. EEM (5w), *p* < 0.01; MQ vs. EEM (6w), *p* < 0.01; MQ vs. EEM (8w), *p* < 0.01; *n* = 5 animals for each group]. **(E)** The hind paw withdrawal thresholds in response to mechanical stimulation after SCI in the different treatment groups [*F*(3, 16) = 169.4, *p* < 0.01. Tukey’s *post hoc* test. MQ vs. EEM (1d), *p* = 0.99; MQ vs. EEM (3d), *p* = 0.54; MQ vs. EEM (7d), *p* < 0.01; MQ vs. EEM (2w), *p* < 0.05; MQ vs. EEM (3w), *p* < 0.01; MQ vs. EEM (4w), *p* < 0.05; MQ vs. EEM (5w), *p* < 0.05; MQ vs. EEM (6w), *p* < 0.05; MQ vs. EEM (8w), *p* < 0.05; *n* = 5 animals for each group]. **(F)** Immunofluorescence tracing of PKH26-labeled EEM or MQ in the injured spinal cord at the T10 spinal segment area: F4/80 (green), PKH26 (red), nuclei DAPI (blue). Scale bar: 200μm. *n* = 3 animals per group. **(G)** Magnification of **(F)**. **(H)** Survival rate of transplanted EEM and MQ by flow cytometry. **(I)** Percentages of PKH26^+^DAPI^–^ /PKH26^+^ cells of **(H)** (*t* = 1.08, *p* = 0.62; *n* = 3 animals per group). **(J)** Representative electrophysiological traces in the different treatment groups. **(K)** Quantification of the amplitude and latency period of MEPs in the different treatment groups [latent period: *F*(3, 16) = 44.20, *p* < 0.01. Tukey’s *post hoc* test. Control vs. MQ, *p* = 0.67; MQ vs. EEM, *p* < 0.05; amplitude of hindlimb: *F*(3, 16) = 236.8, *p* < 0.01. Tukey’s *post hoc* test. Control vs. MQ, *p* = 0.81; MQ vs. EEM, *p* < 0.01; *n* = 5 animals for each group]. The data are presented as the means ± SD. *p < 0.05, **p < 0.01, NS = Not significant.

To explore whether EEM transplantation in the injured spinal cord could improve functional recovery after SCI, we labeled EEM or MQ with DiR (DiR cells) and performed *in vivo* tracking of cell distribution in the injured spinal cord with hydrogel embedding after local transplantation (Figures 4A,B) using the Xenogen IVIS imaging system. The results showed that DiR cell fluorescence accumulated in the injury site of the spinal cord, but no difference in average radiant efficiency between the EEM group and MQ group (Figures 4B,C). In order to further explore the distribution of transplanted EEM and MQ. We mixed EEM or macrophages labeled with PKH26 into hydrogel and applied it on the surface of injured spinal cord. Fluorescence staining demonstrated that both transplanted EEM or macrophages survived in the spinal cord and were mainly distributed in the lesion area (Figures 4F,G). To evaluate the survival of transplanted cells, we applied F4/80 antibody to sort out the macrophages of the spinal cord using flow cytometry at 7 days after injury. The double-positive cells for PKH26 and F4/80 cells were the transplanted macrophage cells. DAPI was used to identify live and dead cells. Under this condition, the proportion of PKH26^+^DAPI^+^ cells to total PKH26^+^ cells could define the survival rate of sorted–transplanted cells. The survival rates of sorted–transplanted cells in both the EEM and MQ grafted group were approximately 90%, but no difference was found between these two groups (Figures 4H,I). We also found that EEM transplantation could reduce the expression of pro-inflammatory factors (iNOS, TNF-α, and CCR7) while increasing the expression of inhibitory inflammatory factors (Arg-1, CD206, and IL-10) when compared to that of macrophage transplantation (Supplementary Figure 1). Therefore, these results indicated that EEM transplantation provided an inflammation–inhibitory microenvironment.

After EEM treatment, mice in the injury group exhibited improved neurological functional recovery compared with those in the MQ treatment group, as indicated by the BMS scores (Figure 4D). For the sensory improvement test, the withdrawal thresholds in response to mechanical stimuli decreased significantly in EEM-treated mice over time after SCI compared with MQ-treated mice (Figure 4E). Moreover, electrophysiological analysis demonstrated that the amplitudes of motor-evoked potentials (MEPs) increased significantly after EEM treatment, and the latent period decreased significantly in EEM-treated mice compared with macrophage-treated mice at 56 days post SCI (Figures 4J,K). Collectively, these findings suggested that EEM could improve sensory and locomotive function after SCI.

### Exosome-Educated Macrophages Promote Axon Growth and Angiogenesis After Spinal Cord Injury

**FIGURE 5:**
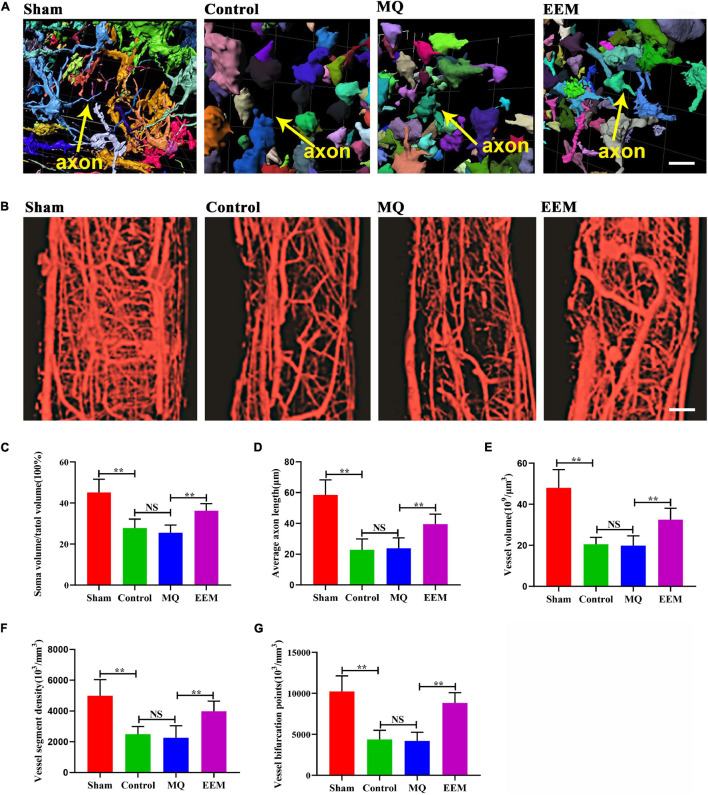
EEM promote 3D morphological changes in spinal cord neurovascular network after SCI. **(A)** Representative 3D morphological image of spinal cord neuronal network changes including the presumptive fide axons among the different treatment groups. Scale bar: 10 μm. **(B)** Representative 3D images of the spinal cord microvasculature in the different treatment groups. Scale bar: 100 μm. **(C)** Percentage of soma volume to total volume in **(A)** [*F*(3, 20) = 22.23, *p* < 0.01. Tukey’s *post hoc* test. Control vs. Sham, *p* < 0.01; MQ vs. Control, *p* = 0.82; MQ vs. EEM, *p* < 0.01; *n* = 6 animals for each group]. **(D)** Average axon length in **(A)** [*F*(3, 20) = 28.74, *p* < 0.01. Tukey’s *post hoc* test. Control vs. Sham, *p* < 0.01; MQ vs. Control, *p* = 0.99; MQ vs. EEM, *p* < 0.01; *n* = 6 animals for each group]. **(E)** Quantification of vessel volume in **(B)** [*F*(3, 20) = 29.02, *p* < 0.01. Tukey’s *post hoc* test. Control vs. Sham, *p* < 0.01; MQ vs. Control, *p* = 0.99; MQ vs. EEM, *p* < 0.01; *n* = 6 animals for each group]. **(F)** Quantification of vessel segment density in **(B)** [*F*(3, 20) = 16.51, *p* < 0.01. Tukey’s *post hoc* test. Control vs. Sham, *p* < 0.01; MQ vs. Control, *p* = 0.95; MQ vs. EEM, *p* < 0.01; *n* = 6 animals for each group]. **(G)** Quantification of vessel bifurcation in **(B)** [*F*(3, 20) = 30.05, *p* < 0.01. Tukey’s *post hoc* test. Control vs. Sham, *p* < 0.01; MQ vs. Control, *p* = 0.99; MQ vs. EEM, *p* < 0.01; *n* = 6 animals for each group]. The data are presented as the means ± SD. *p < 0.05, **p < 0.01, NS = Not significant.

**FIGURE 6:**
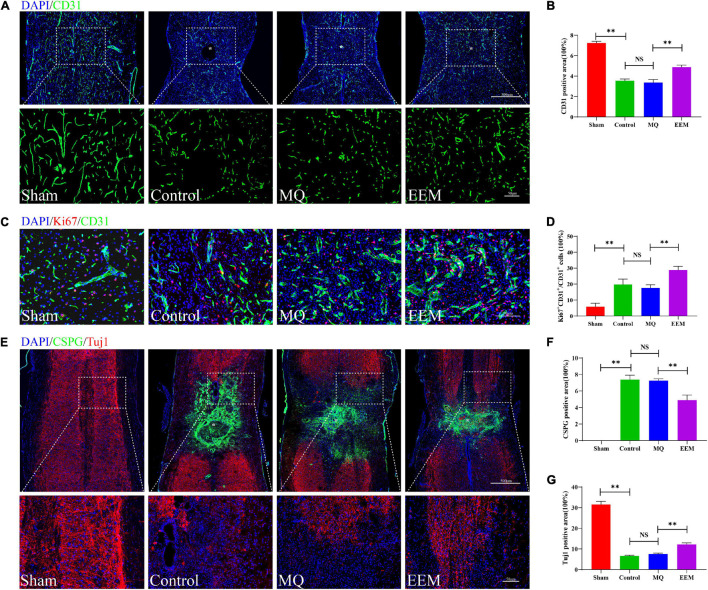
EEM promote angiogenesis and axon growth after SCI. **(A)** Representative immunofluorescence images of CD31 (green) in the different treatment groups. Scale bars: 500μm (upper panel), 50 μm (lower panel). **(B)** Quantification of CD31-positive areas in **(A)** [*F*(3, 12) = 276.1, *p* < 0.01. Tukey’s *post hoc* test. Control vs. Sham, *p* < 0.01; MQ vs. Control, *p* = 0.63; MQ vs. EEM, *p* < 0.01; *n* = 6 animals for each group]. **(C)** Representative immunofluorescence images of CD31 (green) and Ki67 (red) in the different treatment groups. Scale bars: 50?μm. **(D)** Quantification of ratio of CD31^+^Ki67^+^/CD31^+^ cells in **(C)** [*F*(3, 12) = 53.98, *p* < 0.01. Tukey’s *post hoc* test. Control vs. Sham, *p* < 0.01; MQ vs. Control, *p* = 0.63; MQ vs. EEM, *p* < 0.01; *n* = 6 animals for each group]. **(E)** Representative immunofluorescence staining of Tuj-1 (red) and CSPG (green) in different treatment groups. Nuclei were stained with DAPI (blue). Scale bars: 500μm (upper panel), 50 μm (lower panel). **(F)** Quantification of CSPG-positive areas in **(E)** [*F*(3, 12) = 265.2, *p* < 0.01. Tukey’s *post hoc* test. Control vs. Sham, *p* < 0.01; MQ vs. Control, *p* = 0.98; MQ vs. EEM, *p* < 0.01; *n* = 6 animals for each group]. **(G)** Quantification of Tuj-1 positive areas in **(E)** [*F*(3, 12) = 665, *p* < 0.01. Tukey’s *post hoc* test. Control vs. Sham, *p* < 0.01; MQ vs. Control, *p* = 0.49; MQ vs. EEM, *p* < 0.01; *n* = 6 animals for each group]. The data are presented as the means ± SD. *p < 0.05, **p < 0.01, NS = Not significant.

The 3D neuronal and vascular networks of the spinal cord are shown in Figure 5. The 3D morphology of soma and neurite were well visualized and comprehensively analyzed (Figure 5A). However, it is not possible to distinguish axons from dendrites in SCI lesion conditions. Thus, in Figure 5A, the neurite was presumptive axons/dendrites. The 3D morphology of the spinal cord microvasculature is perfectly delineated in Figure 5B. Based on the 3D image obtained using SRμCT, we revealed that the neurovascular structure was severely damaged after SCI (Figures 5A,B). After 3D quantitative analysis, we demonstrated that the neuronal soma volume fraction and axon length in EEM-treated mice were greater than those in mice treated with MQ at 56 days after SCI (Figures 5C,D). In addition, 3D vascular parameters, including the vessel volume fraction (VVF), vessel segment density (VSD), and bifurcation point density (BPD), were tested in the EEM-treated group and significantly increased in contrast to the values of the MQ-treated group 56 days after SCI (Figures 5E–G). To validate the results of the SRμCT image analysis, we conducted 2D immunofluorescence staining post SCI in response to treatment with EEM or MQ (Figure 6). Immunofluorescence staining for CD31 (Figure 6A) and Tuj-1 (Figure 6E) was performed separately. For axon statistics, the picked ROI area was 500 μm away from the injury center. The CSPG-positive area was calculated for scar area evaluation, as previously described (Li X. et al., 2020). For vessel densities measurement, we calculated the CD31-positive area containing the injury area according to a previous research (Ni et al., 2019). The white star indicates the injury center. The quantitative results showed that the CD31- and Tuj-1-positive areas were significantly increased in the EEM treatment group compared with the MQ group or control group (Figures 6B,G). We co-stained CD31 with Ki67 to evaluate proliferation of spinal cord microvascular endothelial cells (SCMECs), as shown in Figure 6C. Ki67^+^CD31^+^ cells were obviously increased in the EEM group compared with the MQ group or control group (Figure 6D). Chondroitin sulfate proteoglycans (CSPG) is an important endogenous inhibitor for axon regrowth (Rosenzweig et al., 2019), and EEM transplantation significantly reduced the CSPG-positive area (Figures 6E,F). Collectively, these results indicated that the administration of EEM was beneficial for axon growth and angiogenesis in an acute SCI mouse model.

## Discussion

**FIGURE 7:**
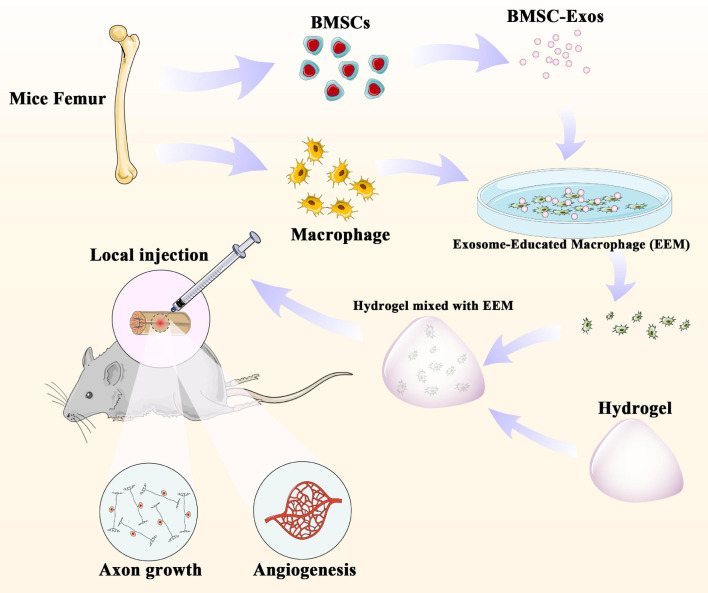
BMSC-derived exosomes extracted from mouse femurs were cultured with macrophages to obtain exosome-educated macrophages (EEM). EEM and hydrogel were mixed and applied to the surface of the injured spinal cord to promote axonal regeneration and angiogenesis.

In our present study, we first examined the effect of the administration of EEM on neurological functional recovery after SCI. Our results demonstrate that the direct application of EEM could improve the neurological functional recovery by promoting angiogenesis coupled with axon growth. These findings shed light on the importance of the application of EEM for neurological recovery and could be a novel therapeutic strategy for SCI treatment (Figure 7).

Macrophages are present in all tissues in the human body and are essential for tissue repair (Cattin et al., 2015). Macrophages can sense the microenvironment and exhibit highly plastic phenotypes (Oishi and Manabe, 2018; Xie et al., 2019). The importance of macrophages during the healing process has rarely been considered or taken into account, except in the context of macrophage depletion (Cattin et al., 2015). The functions of activated macrophages within the CNS are complicated. Recently, a series of studies have explored the effects of direct transplantation of activated macrophage on the regeneration process. Activated macrophage transplantation in the injured spinal cord can simultaneously promote axonal regeneration and release factors that kill cells (Gensel et al., 2009). Studies have also reported the beneficial roles of macrophages in promoting remyelination and the clearance of inhibitory myelin debris (Rawji et al., 2016). Adoptive transfer of M2 macrophages into the injured spinal cord could create a beneficial microenvironment to rescue residual myelin and improve neurological functional recovery after SCI (Shechter et al., 2013). The administration of M2 macrophages could terminate neuroinflammation and ameliorate cognitive dysfunction in amyloid-β-induced rats (Zhu et al., 2016).

In our current study, transplanted macrophages were first educated with exosomes derived from BMSCs, rather than directly transplanted into the injured spinal cord, and these educated macrophages could facilitate healing after SCI by promoting angiogenesis and axon growth. Neurovascular units (NVUs) in the spinal cord are multicellular complexes consisting of endothelial cells, pericytes, and neurons (Xu et al., 2017). Within the NVU, neurons closely associate with the remodeling vasculature. SCI results in disruption of the NVU structure (Xu et al., 2017). Clinical evidence indicates that promoting angiogenesis in the injured spinal cord is beneficial for functional recovery after SCI (Zhou et al., 2019). The generation of new vasculature within the injured spinal cord provides blood support for neuronal survival and facilitates highly coupled neurogenesis, which in turn leads to improved neurological functional recovery (Hu et al., 2015; Hesp et al., 2018). Angiogenesis was associated with neuroprotection by providing nutrients for neuro tissue (Fassbender et al., 2011). In addition, the vasculature may act as a scaffold and guide axonal sprouting after injury (Oudega, 2012). There is also increasing evidence demonstrating that to accelerate regeneration after SCI, the promotion of neurogenesis must be coupled with angiogenesis (Yu et al., 2016). In our study, EEM was an ideal approach and has the capacity to enhance angiogenesis coupled with axon growth after SCI, which associates with improved neurological functional recovery. However, the specific factors secreted from EEM that induce angiogenesis and axon growth in the injured spinal cord are largely unknown. Future studies are needed to understand the molecular mechanisms by which EEM promote spinal cord healing. We will next examine protein expression in CM isolated from EEM.

Despite promising results in some preclinical trials, BMSCs have not been considered standard cell therapies in clinical translation (Musiał-Wysocka et al., 2019), and EEM are terminally differentiated cells that will not differentiate into other cell types, which is a concern associated with BMSCs. Moreover, macrophages were educated with exosomes derived from BMSCs rather than directly cocultured with BMSCs, indicating that BMSCs promote macrophage polarization *via* a paracrine effect through the secretion of extracellular vehicles (EVs) (Kim and Hematti, 2009; Liu et al., 2020). Among various EVs, exosomes are of great interest and currently have several attractive characteristics (Zhang et al., 2019). Exosomes carry cell-specific cargos such as proteins, lipids, mRNAs, microRNAs, and tRNAs to target cells and reprogram recipient cells upon selective uptake (Zhang et al., 2019). The ability of EEM to enhance tendon healing is well researched, but this is the first study to apply this type of therapeutic approach for SCI repair. Our results demonstrated that MSC-derived exosomes promote the switching of recipient macrophages toward the M2-like phenotype *in vitro*, bypassing the indirect effect of MSCs on *in situ* macrophages and providing us with a novel therapeutic approach in the regeneration field.

Since spinal cord neurovasculature has unique 3D morphological features (Li P. et al., 2020; Li R. et al., 2020; Jiang et al., 2021a), we performed a comparative analysis of the vasculature and neuronal network between control and EEM-treated mice and examined changes in the 3D morphological parameters of the spinal cord neurovascular structure after SCI using SRμCT. SRμCT is now routinely used to perform investigations on a wide range of biospecimen. The wide beam provided by the synchrotron radiation allows 3D imaging of large objects at high resolution. The x-ray light coherence and monochromaticity provided at synchrotron experimental beamline stations enable high sensitivity to weakly absorbing tissue. It permits imaging of soft tissue such as the vasculature visualization without contrast agents’ perfusion. Our research group has already started to apply SRμCT to visualize the vascular and neuronal networks in a rat model of chronic compressive thoracic SCI (Jiang et al., 2020). This method enables visualization of the 3D structure of the entire neural and vascular network of the spinal cord. 3D computational analysis of hierarchical images allows for the characterization of the vascular and neural networks to obtain various quantitative parameters. The 3D results will enhance our understanding of the pathophysiology after SCI and provide a new platform to evaluate the effect of EEM on neurovascular regeneration after SCI.

Collectively, the current experimental work demonstrated with the effect of a novel type of macrophage on healing after SCI. Indeed, we could not exclude the direct or indirect effect of EEM on neurological functional recovery after SCI. The biological and neurological functional recovery reported herein indicates that EEM could promote angiogenesis and axon growth and create a regenerative environment for SCI healing. These results provide an alternative approach of using educated macrophages to alleviate SCI and may have wide applications in CNS disorders.

## Data Availability Statement

The original contributions presented in the study are included in the article/Supplementary Material, further inquiries can be directed to the corresponding author/s.

## Ethics Statement

The animal study was reviewed and approved by the Animal Ethics Committee of Central South University, Changsha, China.

## Author Contributions

JH, YC, and HL designed and supervised the study. CL, TQ, RH, and JZ did the experiment. YC, CL, TQ, CD, and HW analyzed the data. YC and CL wrote and revised the manuscript. All authors contributed to the manuscript.

## Conflict of Interest

The authors declare that the research was conducted in the absence of any commercial or financial relationships that could be construed as a potential conflict of interest.

## Publisher’s Note

All claims expressed in this article are solely those of the authors and do not necessarily represent those of their affiliated organizations, or those of the publisher, the editors and the reviewers. Any product that may be evaluated in this article, or claim that may be made by its manufacturer, is not guaranteed or endorsed by the publisher.
