# Supplementary material for: Bone Marrow Mesenchymal Stem Cell-Derived Exosome-Educated Macrophages Promote Functional Healing After Spinal Cord Injury
Source: Front Cell Neurosci. 2021 Sep 28;15:725573. doi: 10.3389/fncel.2021.725573 (PMC8506031; doi:10.3389/fncel.2021.725573)
Supplement: Supplementary file 1 [file Presentation_1.pdf]

Supplementary data

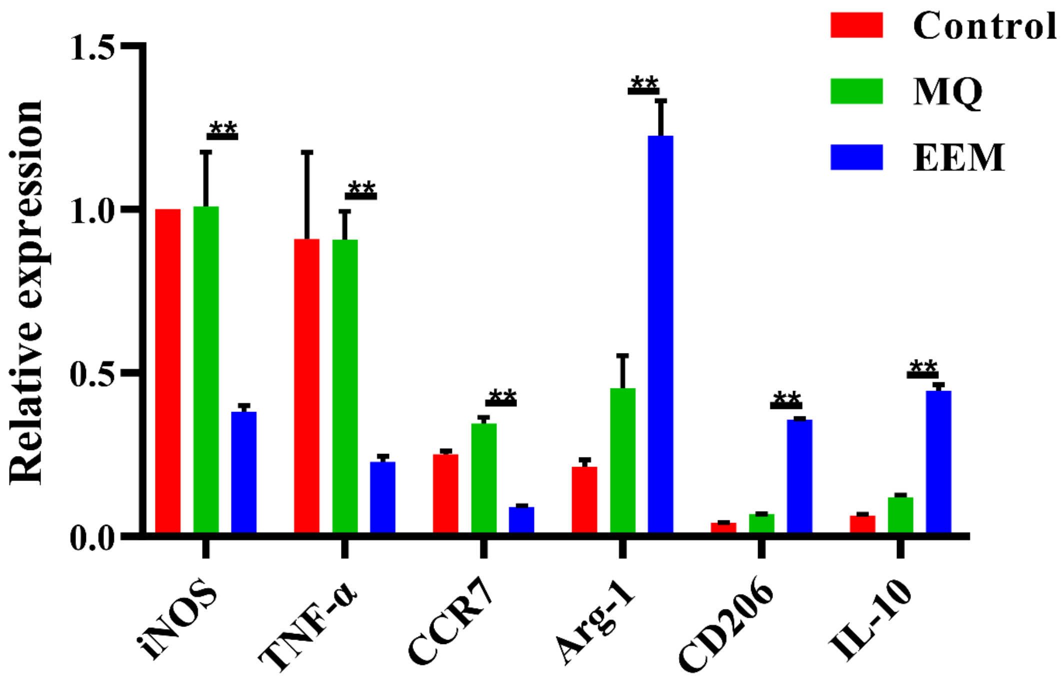

Figure S1 Expression of pro-inflammatory factors (iNOS, TNF-α, CCR7) and inhibitory inflammatory factors (Arg-1, CD206, IL-10) by qRT-PCR (iNOS: t=6.52, $p < 0.01$; TNF-α: t=13.35, $p < 0.01$; CCR7: t=22.78, $p < 0.01$; Argnase-1: t=9.17, $p < 0.01$; CD206: t=12.56, $p < 0.01$; IL-10: t=28.26, $p < 0.01$; n=3 replicates for each group). The data are presented as the means ± SD. **$p < 0.01$.
